# Objective Assessment of an Ionic Footbath (IonCleanse): Testing Its Ability to Remove Potentially Toxic Elements from the Body

**DOI:** 10.1155/2012/258968

**Published:** 2011-11-29

**Authors:** Deborah A. Kennedy, Kieran Cooley, Thomas R. Einarson, Dugald Seely

**Affiliations:** ^1^Department of Research & Clinical Epidemiology, The Canadian College of Naturopathic Medicine, 1255 Sheppard Avenue East, Toronto, ON, Canada M2K 1E2; ^2^Leslie Dan Faculty of Pharmacy, University of Toronto, 144 College Street, Toronto, ON, Canada M5S 3M2; ^3^Clinical Epidemiology Program, Ottawa Hospital Research Institute, 501 Smyth Road, Box 201, Ottawa, ON, Canada K1H 8L6

## Abstract

Ionic footbaths are often used in holistic health centres and spas to aid in detoxification; however, claims that these machines eliminate toxins from the body have not been rigorously evaluated. In this proof-of-principle study, we sought to measure the release of potentially toxic elements from ionic footbaths into distilled and tap water with and without feet. Water samples were collected and analyzed following 30-minute ionic footbath sessions without feet using both distilled (*n* = 1) and tap water (*n* = 6) and following four ionic footbaths using tap water (once/week for 4 weeks) in six healthy participants. Urine collection samples were analyzed at four points during the study. Hair samples were analyzed for element concentrations at baseline and study conclusion. Contrary to claims made for the machine, there does not appear to be any specific induction of toxic element release through the feet when running the machine according to specifications.

## 1. Introduction

With the advent of the industrial revolution, the levels of toxicants in our water, air, and soil have risen dramatically such that even newborn infants are born with toxic elements and chemical pollutants in their bodies [1]. There are a host of illnesses attributed to toxin exposure that have arisen in the 20th century that were not previously recognized. Sick building syndrome and multiple chemical sensitivity are attributed, in part, to bioaccumulation of toxins and pollutants [2]. As well, the rate of increase in cancers is greater for those born after 1940 [2, 3]. While causative links are difficult to prove, it is hypothesized that the burden of toxic elements is linked to a number of health conditions including mental health [4], ADHD [5], cancer [3, 6–9], reproductive health [10, 11], and autoimmune conditions [12]. 

Currently, many methods of detoxification are available, such as dimercaptosuccinic acid (DMSA), which is known to bind to heavy metals and aid in their elimination from the body [13–15]. Infrared and dry heat saunas can also detoxify, partly by breaking down body fat which liberates fat-soluble substances, medications, and heavy metals stored in adipose tissue [16, 17]. More recently, ionic footbaths have been promoted as a means of eliminating toxins and heavy metals from the body in the lay literature and worldwide web [18]. 

Consumer use of ionic footbaths appears to come predominantly from holistic health centres, hair salons, and health food stores which often promote ionic footbaths as a means to rid the body of toxins such as heavy metals and often charge upwards of $75 per session [19–21]. 

Following an empty search of Medline, EMBASE, AMED, Alt Health Watch, and CINAHL using the search terms “ionic,” “footbath,” and “detoxification,” a search on Google found one study conducted by the Centre for Research Strategies [22]. That study found a statistically significant reduction in aluminum and arsenic, but no changes in lead, mercury, or cadmium in whole blood of the participants after 12 weekly sessions [22]. Concomitant nutrition and meditation techniques were used, making the contribution from footbaths impossible to isolate. In addition there was a risk of bias for this study demonstrated by poor quality reporting (12-week results reported only yet the protocol described a 6-month study), a lack of scientific rigor in the methods, and potential for conflict of interest, the research was conducted by “The Centre for Research Strategies,” an arm of the IonCleanse manufacturer. Unbiased, reliable information on prevalence of consumer use, as well as scientific investigation of the methods and purported effects of these devices, remains scarce.

In this proof-of-principle study, we evaluated the IonCleanse Solo footbath. This product has been available in the market since 2002 [18] and has successfully undergone electrical appliance safety testing. It received both Federal Communications Commission** (**FCC) and Conformité Européenne (CE) approvals [18, 23–25]. 

This was a two-phase project. The objective of Phase I was to establish a baseline for the contribution of the ionic footbath machine to release potentially toxic elements (PTEs) when either distilled or tap water was used without feet present. Phase II had several objectives including whether the ionic footbath could (1) effectively remove PTEs through the feet of participants; (2) increase PTE release through the urine; (3) increase PTE release as measured through hair mineral analysis (HMA).

## 2. Materials and Methods

### 2.1. Study Design

This was a proof-of-principle, nonrandomized, nonblinded comparative, no feet versus feet, trial conducted from the week of May 17, 2010 (Week 0) through to August 9, 2010 (Week 12). Ethics approval was given by Research Ethics Board of the Canadian College of Naturopathic Medicine (CCNM) according to the ethical standards set forth in the 1975 Helsinki Declaration. All participants enrolled gave written informed consent to participate in the study. This study was funded through a grant from the Holistic Health Research Foundation. The trial registry number is NCT01125592.

#### 2.1.1. Participants

Between April and May 2010, healthy participants were recruited through e-mail to CCNM staff and students, website-based advertisements, and posters. The e-mail summarized the requirements for the study and asked interested individuals to respond to the study coordinator. The study was also open to the general public.

Inclusion criteria required participants over 18 years of age, in good health, and with a stable medication/supplementation regimen for at least six weeks prior to and during participation in the study. Individuals were excluded if they were not legally competent; were pregnant or nursing mothers; had a pacemaker; were organ transplant or metal joint implant recipients; took antiarrhythmic, anticoagulant or chelating medication; or took any medication whose absence could mentally or physically incapacitate them (antipsychotics, antiepileptics, etc.). Participants were excluded if they had used a sauna within two weeks prior to beginning the study. Participants were also instructed to avoid sauna use during the study. 

#### 2.1.2. Ionic Footbath Device

**Figure 1:**
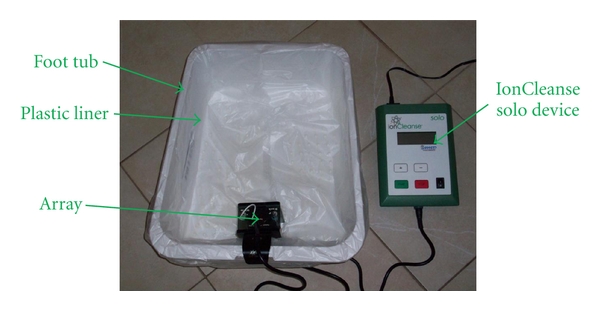
Initial setup of IonCleanse SOLO footbath.

IonCleanse SOLO (A Major Difference Inc., Aurora, Colo) ionic footbath was used for all sessions in the study. With knowledge of the trial to be conducted, A Major Difference Inc. donated an IonCleanse SOLO machine for the duration of this study. The components of the ionic footbath include the SOLO device, an array, a power cord, plastic foot tub liners, and a plastic foot tub container (Figure 1). The SOLO device has a single preset program to generate a 70/30 mix of positive/negative polarity in a standard 30-minute session. 

**Figure 2:**
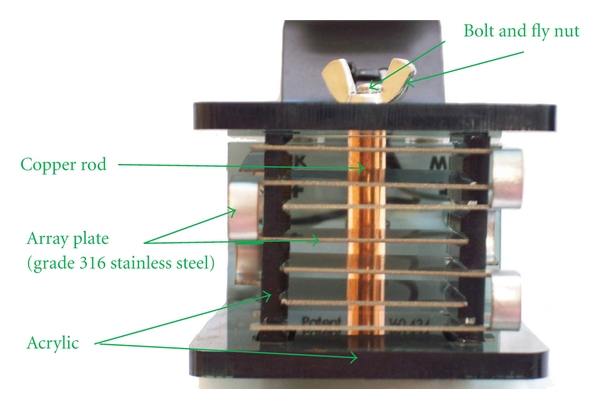
Close up of a new IonCleanse SOLO footbath array.

The array is composed of an acrylic housing, a copper rod held in place with a bolt and fly nut, and a metal plate folded on itself several times (Figure 2) [18]. The side of the array is stamped with “316 SS” which we interpreted to indicate that the metal is composed of “316 grade stainless steel.” The metal plates of the array have a limited lifespan and must be replaced after 30–50 sessions, with the “life” of a metal plate dependent on the mineral concentration of the water source [18]. 

#### 2.1.3. Setup and Running of the Footbath Device

The IonCleanse SOLO footbath was set up according to manufacturer's instructions as follows. A new plastic liner lined the foot tub and the “source” water was used to fill the foot tub (approximately 3.75 litres of water per session). The array was plugged into the SOLO device and placed in the foot tub, ensuring that there was sufficient water to cover the copper bar of the array. The device was turned on, and both voltage and amperage, displayed on the front of the machine, were monitored to ensure they stayed within optimal operating range, 13–20 volts and 1.8–2.2 amperes, respectively. This range was maintained for all footbath sessions, and no changes were made to the preset program on the SOLO device. Each session ran for 30 minutes, indicated by a buzzer at the end of the session.

### 2.2. Setting

All footbath sessions were conducted at the Robert Schad Naturopathic Clinic (RSNC) located within CCNM.

### 2.3. Phase I: Establishment of Baseline and Potential Confounders

**Distilled Water Procedure**

Three independent footbath sessions using two brands of distilled water (Life Brand and Longo's, 4-litre plastic container, steam-distilled water) were run. A sample of the distilled water was placed in the 100 mL sample bottle and labelled. The footbath was prepared as described above using distilled water. The machine was turned on, and 1/8 tsp of salt (Baleine Sea Salt, 30220 Aigues-Mortes, France), according to the manufacturer's instructions, was placed in the footbath water. At the end of the session, the water was stirred and a sample taken. This procedure was used for the first two footbath sessions with distilled water. In the last session, the sample was obtained after the salt had been added to the foot tub. The footbath session continued as described above.

**Tap Water Baseline and Postsession Procedures**

The following procedure was used for all tap water footbath sessions. At the outset, it was determined that 50 L of water would be required to conduct the six footbath sessions. A 105 L plastic container (Storage Solutions, Gracious Living, Woodbridge, ON) was used for all tap water tests. The level of 50 L was predetermined and marked on two of the outside walls of the 105 L plastic container. The hot and cold tap water was run for 30 seconds to ensure that no stagnant water remained in the pipes. The 105 L container was filled to the predetermined level with a mixture of hot and cold water, and, according to the manufacturer's instructions, 6 × 1/8 tsp of sea salt (Baleine Sea Salt, 30220 Aigues-Mortes, France) were added and stirred 20 times. A water temperature of approximately 39-40°C was used. A 100 mL sample of the tap water was obtained and labelled with the identifier “CCNM” and a sequential number. The samples were numbered in the sequence in which they were obtained to blind the laboratory to the source of the water sample. Samples were placed in the refrigerator overnight and couriered to the laboratory the following day.

On Week 0, the baseline parameters of the footbath device were established as follows: daily, for three consecutive days, the SOLO device was set up as before and run for 30 minutes with no feet in the footbath water. Samples were taken. On Week 5, after all participant footbath sessions had been completed, three additional postsession “no feet” sessions were conducted on the same day and samples obtained.

### 2.4. Phase II: Assessment for Efficacy in Removal of Potentially Toxic Elements

**Figure 3:**
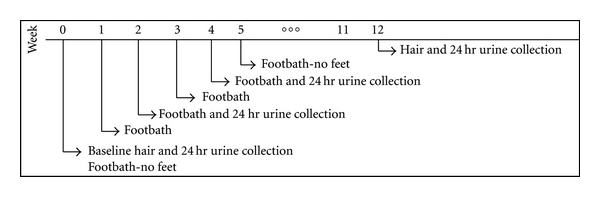
Study schedule.

An overview of the study schedule is provided in Figure 3.

**Establishment of Baseline and Postsession Parameters for Participants**

At baseline and Week 12, participants were requested to provide a hair and 24-hour urine sample for analysis following instructions provided by the laboratory for obtaining these samples. Hair is a very stable medium [41] and therefore regular mail (Letter, Canada Post, Ottawa, Canada) was used to send the hair samples in sealed envelopes to the laboratory for analysis. Participants were instructed to obtain their second hair sample from the same location as the first, so that the second sample better represented what had been circulating in the blood during the previous 3-month period. For the urine samples, participants were provided with courier forms and packaging materials and asked to contact the courier company (Xpresspost, Purolator, Mississauga, ON) for shipment pickup and overnight delivery to the laboratory.

**Assessment of Detoxification through Urine**

Twenty-four-hour urine collections were also collected during the 24 hours following the second and fourth footbath sessions. Collection began the day of the footbath session and continued until the first morning void the day after.

**Footbath Sessions with Participants**

Footbath sessions were scheduled weekly on the same weekday and time. To decrease any residual particulate matter or mineral-containing excretions participant's feet were rinsed under running water prior to placing their feet in the foot tub. The tap water and footbath device were set up as previously described for the initial footbath session. For all footbaths conducted within the day, the 105 L container was used as a consistent water source. Participants placed their washed feet into the prefilled foot tub and the SOLO device turned on. At the end of 30 minutes, participants removed their feet from the footbath, the footbath water was stirred and a sample taken and labelled. At the end of the day, all samples were collected and couriered to the laboratory. The array was removed from the footbath and rinsed with clean water. Once the visible residue was removed, a disinfectant (Ultra-Safe Plus commercial cleaner, Safer Soaps, Traveler's Rest, SC) was sprayed on the array as per the manufacturer's recommendations. Several minutes later, the array was rinsed and dried with a clean towel.

Each week, the array was soaked in a dilute solution of ascorbic acid (A Major Difference Inc., Aurora, Colo) and water according to manufacturer's instructions.

### 2.5. Laboratory Analysis

Water, hair, and urine analyses were performed using Inductively Coupled Plasma Source Mass Spectroscopy (ICP-MS) by CanAlt Health Laboratory Inc., Concord ON, Canada. Calibration of the method has been carried out using at least two internationally recognized National Institute of Standards and Technology (NIST) standards for each element and is validated by analysis of Certified Reference Material (CRM). CanAlt Health Laboratory follows and documents Good Laboratory Practice Standards for handling of materials, quality control, and standardization of instruments to control for determinate error and to provide quality assurance. 

### 2.6. Statistical Analysis

**Water Samples**

The water reports provided by CanAlt Health Laboratory Inc. list the concentrations of 28 individual elements. Descriptive statistics (total, mean, standard deviation) were calculated for each element. In addition, to facilitate reporting, elements tested were categorized into three groups and subtotals determined for “array components,” “essential elements,” and “PTEs” (Table 1).

The change in each element's concentration was calculated by subtracting the concentration in the postfootbath session (Post-FBS) from the concentration in the source sample (Pre-FBS) to derive the difference (Diff-FBS). There were 3 distinct groups of water samples: (1) distilled water with no feet, (2) tap water with no feet, and (3) tap water with feet. Mann-Whitney tests compared the Post-FBS to the Pre-FBS element concentration to determine whether the Diff-FBS element concentration was statistically significant. This analysis was done for both the tap water with no feet and the tap water with feet groups. One valid observation was sufficient for the highly controlled distilled water source to act as a comparison group, and this precluded use of the Mann-Whitney test. Also, Mann-Whitney test compared the Diff-FBS (no feet/feet) to determine whether the presence of participants' feet affected results. A Kruskal-Wallis test comparing the total concentrations of the Pre-FBS and Post-FBS tap water with no feet and Post-FBS tap water with feet was used to determine whether a significant difference existed between the groups.

**Hair Mineral Analysis (HMA)**

HMA reports list the concentration of 40 individual elements. Total PTEs, defined as Al, Sb, As, Ba, Beryllium (Be) [26, 42, 43], Cd, Mercury (Hg) [26, 44–46], Pb, and U, were summed for HMA results.

**Urine Analysis (UA)**

The UA reports list the concentrations of 40 individual elements. Total PTEs, defined as Al, Sb, As, Ba, Be, Cd, Hg, Pb, and U, were summed for UA results. Microsoft Office Excel-2007 was used for all data manipulations and descriptive statistics. StatsDirect version 2.7.7 was used for the nonparametric statistics.

## 3. Results and Discussion

**Participants**

An e-mail request was sent out to all the staff (*n* ~ 100) at the CCNM to solicit possible recruits. The first participants who responded were assessed for eligibility leading to three people excluded due to (1) an inability to commit to the schedule of footbaths; (2) not able to maintain a stable medication/supplement regime; (3) presence of a metal implant.  Table 2 summarizes the characteristics of the six study participants that were included. Participants received no compensation for involvement in the study but were provided with copies of the results from their laboratory tests.

While participants' schedules necessitated some minor adjustments of appointment times, all but one of the footbath sessions occurred on the same weekday between 10 AM and 4 PM. One participant's second footbath was performed two days after the usually scheduled session due to an illness unrelated to the study. Participants were requested to maintain a stable lifestyle and medication/supplementation regime throughout; however one participant, during Week 3, needed to take antibiotics for 11 days for an illness unrelated to the study.

The footbath sessions were well tolerated by all of the participants. There were no adverse events reported during the course of the study.

### 3.1. Phase I

#### 3.1.1. Footbath Sessions without Feet Using Distilled Water as Source (*n* = 4) (Table 3)

Though two different sources were used, it is evident from these results that Al, Cu, Fe, and Na were present in the distilled water in small amounts at the outset. In the Post-FBS, the largest changes in element concentrations were for Cr, Co, Cu, Fe, Mn, Mo, Ni, and Si. Total PTEs increased 17 *μ*g/L after running the machine with greatest increases in Al, Sb, As, and Cd. 

#### 3.1.2. Footbath Sessions without Feet Using Tap Water as Source (*n* = 6) (Table 4)

The concentration of essential elements predominates in the tap water prior to the footbath. There are also PTEs in the tap water, with Al representing the largest concentration. In the Post-FBS, as with the distilled water results, the largest changes in element concentrations occur within the array elements (*P* = 0.010). Mean total PTE concentrations also increased by 30.50 *μ*g/L (*P* = 0.133) with nonsignificant increases in Al, Ba, and Pb and significant increases in Sb (*P* = 0.038), As (*P* = 0.010), and Cd (*P* = 0.010). 

### 3.2. Phase II

#### 3.2.1. Footbath Sessions with Feet Using Tap Water as Source (*n* = 24) (Table 5)

The concentration of essential elements (98.9%) vastly outweighs that of PTEs (<1%) in the tap water prior to the footbath. Although present in very low quantities, Al had the highest concentration of all of the PTEs present in baseline tap water. Statistically significant differences were found in Diff-FBS for both array components (*P* < 0.0001) and toxic elements (*P* = 0.042).

We also compared the change in element concentrations (Diff-FBS in tap water with feet versus Diff-FBS in tap water without feet, Table 5). The increase in As was found to be significantly different (*P* = 0.016); however, the differences in total PTE concentration were not (*P* = 0.869), indicating that addition of a person's feet did not significantly alter PTE composition of the water. 

**Figure 4:**
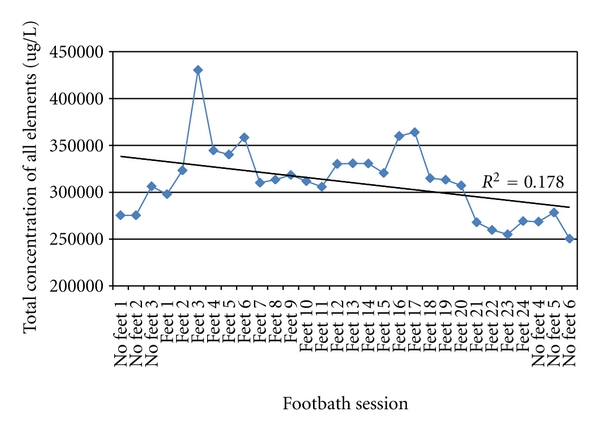
Post-footbath session: total concentration of all elements in order of session occurrence.

**Figure 5:**
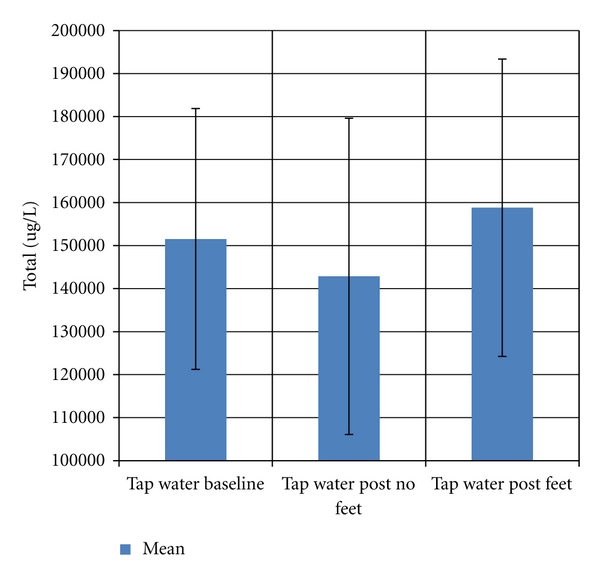
Comparison of mean total PTEs^§^ (*μ*g/L) in tap water: baseline versus Post-FBS no feet versus and Post-FBS with feet. **Error bars represent ± standard deviation from the mean. **^§^**Potentially toxic elements (PTEs) were defined to be aluminium, antimony, arsenic, barium, cadmium, lead, silver, and uranium. **Kruskal-Wallis test found no difference between the three groups (*P* = 0.524).

To assess leeching as a factor in the change of concentration of elements, we plotted the total element concentration in *μ*g/L from Post-FBS in sequence (Figure 4). More elements are discharged into the water when the array is new versus after 40+ sessions (*R* ^2^ = 0.178).  Figure 5 graphically represents the average total elements concentration in *μ*g/L in three groups of results using tap water: Pre-FBS, Post-FBS without feet, and Post-FBS with feet. The Kruskal-Wallis test found no significant differences between the three groups (*P* = 0.524).

#### 3.2.2. 24 Hour Urine Analysis

**Figure 6:**
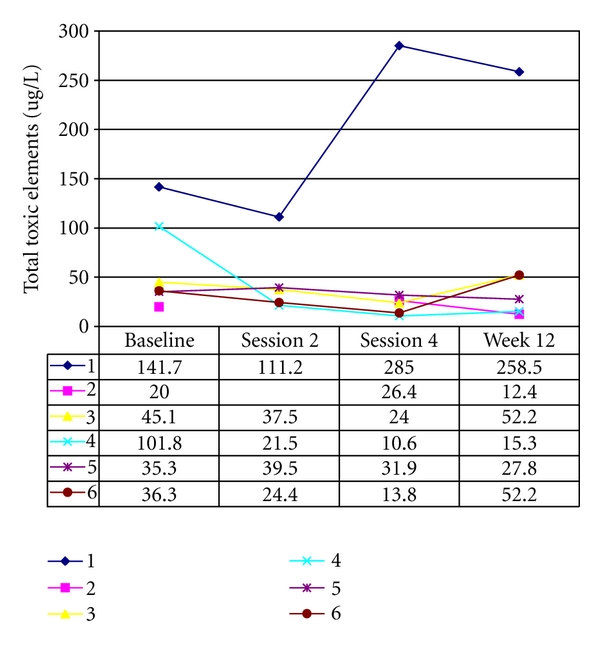
Total PTEs^§^ excreted in urine for each participant. ^§^Total potentially toxic elements (PTEs) were defined to include aluminium, antimony, arsenic, barium, beryllium, cadmium, mercury, lead, and uranium.

Four samples were obtained from the participants: at baseline (Week 0), during the second (Week 2) and fourth (Week 4) footbath sessions, and Week 12 (Figure 3). The total PTEs (Hg, Pb, Al, Cd, Sb, As, Ba, Be, and U) excreted by each participant were graphed (Figure 6). Elimination of PTEs was substantially higher in Participant-1 overall, with initially a reduction in the second footbath followed by an increase in PTE elimination during fourth footbath. Baseline elimination of PTEs was highest for Participant-4 and remained low for each subsequent sample. For the remaining participants, the elimination of PTEs remained stable during the course of the study. The second urine sample for Participant-2 was lost in transit.

#### 3.2.3. Hair Mineral Analysis

**Figure 7:**
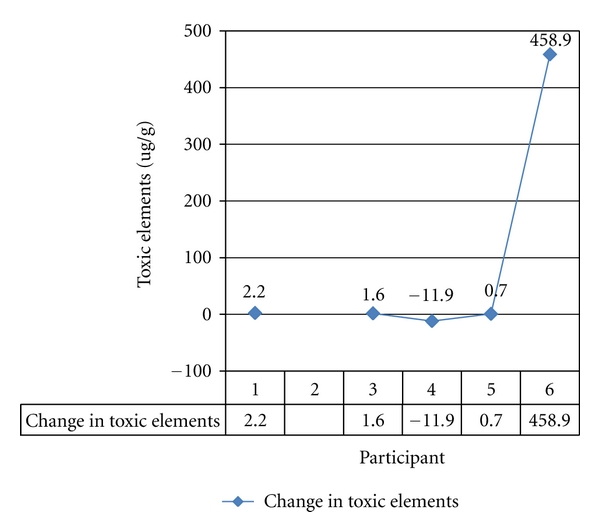
Change in total PTE^§^ (*μ*g/L) in hair: baseline to Week 12. **^§^**Total potentially toxic elements (PTEs) were defined to include aluminium, antimony, arsenic, barium, beryllium, cadmium, mercury, lead, and uranium.

Hair samples were taken at baseline and at Week 12 of the study. PTEs analyzed included Hg, Pb, Al, Cd, Sb, As, Ba, Be, and U. The difference (*μ*g/g) between baseline and Week 12 results of HMA for total toxic elements was graphed (Figure 7). The baseline sample for Participant-2 was lost in transit. For Participant-6, there was a significant change that was highly discrepant from the minimal change in hair PTEs observed for any of the other participants. 

### 3.3. Discussion

We found that the IonCleanse SOLO device did not induce the elimination of PTEs through the feet of study participants. There is no evidence that the device stimulates pathways of PTE elimination through either the kidneys, via urine, or through the hair after receiving four 30-minute footbath sessions given weekly.

#### 3.3.1. Ionic Footbath Effectiveness

The manufacturers of the IonCleanse device claim that their product's effectiveness lies in its ability to generate positively and negatively charged ions (H+, OH−) via electrolysis in water. Purportedly, these ions cause the neutralization and subsequent removal of charged particles from the body via osmosis and diffusion through the skin that is in contact with the ion gradient created in the water. While much attention in the claim is given to the impact this gradient may have on a person whose feet are immersed in this water, little is given towards the impact this gradient may have on the array itself.

Stainless steel is a composite of different elements with Fe as the basic element. The composition of the steel varies, with 316 grade having a higher amount of chromium in order to provide increased resistance to corrosion [48, 49]. The usual composition of 316 grade stainless steel is summarized in Table 7 [48]. The elements with the greatest change in concentration after running the device, with or without feet, were Fe, Cr, Ni, Mo, Mn, and Si. These elements align very closely to those elements common to 316 grade stainless steel. 

Corrosion can be defined as “deterioration of a material due to interaction with its environment. It is the process in which metallic atoms leave the metal or form compounds in the presence of water and gases” [49, 50]. The use of direct current and salt in the water will accelerate the corrosion of the stainless steel. There are PTEs in all of the footbath water Post-FBS regardless of the presence or absence of feet. Sb, As, and Cd were significantly different from the tap water in the Post-FBS without feet sessions; As, Ba, and Cd were significantly different in the Post-FBS with feet sessions. It is difficult to identify the source for the increased elements. Other components of the footbath apparatus represent possible sources. However, since materials analysis of these components was not performed it is difficult to be certain. Regardless, the elevation of PTEs in the sessions without feet strongly suggests that the participants are not the source of PTE elevation in the sessions with feet. This is further supported by the lack of statistically significant change in mean PTEs when with and without feet sessions are compared (Table 6). The overall reduction in total elements present in Post-FBS with each subsequent running of the machine further supports the corrosion idea, as there is less material available to dissociate into the water. 

#### 3.3.2. Elimination through Urine

One hypothesis whereby PTE elimination could be supported using the ionic footbath device is through stimulation of an alternate detoxification pathway through the kidneys. To test this hypothesis, 24-hour urine collections were obtained concurrent with the second and fourth footbath sessions. If the hypothesis was correct, increased elimination resulting in elevated urinary total PTEs in sessions two and four should have been evident over and above baseline. This was not found to be the case. While some variance between participants is evident, during the 4 weeks where participants were receiving footbaths there were no clinically relevant changes in the elimination of PTEs that cannot be differentiated from normal fluctuations in excretion via urinary pathways. It is unclear why results for Participant-1 appeared as an outlier to the general trend in the other participants. Given these results, exposure to four sessions of ionic footbath did not appear to have any substantive influence over the body's ability to eliminate PTEs through the urine.

#### 3.3.3. Hair Mineral Analysis

Hair is a stable medium that records which elements are circulating in the blood, and there is evidence that toxic elements in hair are representative of toxic element levels in the internal organs [51, 52]. Hair grows at the rate of approximately 1 cm per month [41]. Hair also represents a meagre but still possible route of excretion as elements incorporated into the hair shaft are removed from circulation. To test for any changes in PTEs in the hair of participants having the ionic footbath, hair samples for analysis were provided at baseline and Week 12 of the study. We hypothesized that if, because of the ionic footbaths, detoxification pathways related to PTEs were stimulated, there would be elevated levels of these elements in the hair at Week 12 compared to baseline. The difference in toxic elements at Week 12 for all participants but one showed essentially no change. Participant-6's total PTEs at Week 12 was substantially higher over baseline. When compared to Participant-6's urine, the increased level of PTEs in the hair was not offset by a concomitant increase in urinary excretion of toxic elements. The high toxic element findings in the hair may have reflected a redistribution of toxic elements in the body or contamination of the hair sample that we were unable to identify.

#### 3.3.4. Strengths and Limitations

In this trial, we tested the application of the IonCleanse SOLO ionic footbath across the lifespan of an array amongst six individuals. Each participant was exposed to four footbath sessions. It is conceivable that a larger number of sessions are required to see an overall detoxification effect in the individual; however, the lack of observable changes in PTEs in the water that might be attributed to a person seems unlikely. If there was any resistance to effect from a single exposure this was accounted for with multiple exposures over the course of one month.

In addition to testing for possible stimulation of physiological detoxification pathways, we also analyzed pre- and postexposure samples for both urine and hair in each participant. By testing and comparing three possible routes of elimination (feet, urine, and hair) we went beyond the implied claims of direct elimination through the feet by exploring other possible routes of elimination. Budgetary constraints precluded us from examining elimination through the colon as stool. It is possible that detoxification through the liver and bile could have been augmented with exposure. However, as both urinary excretion and HMA did not uncover any significant changes in these routes of elimination over the course of treatment and due to a lack of biological rationale it is unlikely that a liver specific elimination would be stimulated either.

The outcome of primary importance in this study, toxic element concentrations, depends on accurate measurements with low intertest variability. A strength of this study was the quality analysis performed by an independent laboratory following good laboratory practices with expertise in water, urine, and hair mineral analysis. The laboratory was blinded to the source of the water being tested and to the protocol from which sequential participant urine and hair samples were taken. 

This was a proof-of-principle study with a small sample size. The small sample size would not permit us to identify small shifts in the elimination of PTEs through the utilization of the ionic footbath device. It is possible that a larger study may be able to identify clinical significant differences. Further, we tested healthy participants (self-defined and suffering from no major diseases), and it is conceivable that, in people with high levels of toxicity, application of the ionic footbath could have led to increased elimination either directly or indirectly.

We did not perform materials testing on all of the components of the ionic footbath device. As such, we were not able to confirm other potential sources of PTEs that might be contributing to the changes in toxic elements observed between Pre-FBS and Post-FBS without feet. We hypothesized that the elements found in the residual water could come from the array, salt, plastic storage container, or the plastic liner of the foot tub. 

## 4. Conclusions

In this proof-of-principle study we found no evidence to suggest that ionic footbaths help promote the elimination of toxic elements from the body through the feet, urine, or hair. While unlikely to cause harm or result in any increased uptake, the use of ionic footbaths may release minute quantities of PTEs into the aqueous environment.

## Figures and Tables

**Table 1:** Categorization of reported elements by group.

Array components	Essential elements	Potentially toxic elements
(i) Chromium (Cr)	(i) Boron (Bo)	(i) aluminum (Al) [26–28]
(ii) Cobalt (Co)	(ii) Calcium (Ca)	(ii) Antimony (Sb) [26, 29, 30]
(iii) Copper (Cu)	(iii) Lithium (Li)	(iii) Arsenic (As) [26, 29, 31, 32]
(iv) Iron (Fe)	(iv) Magnesium (Mg)	(iv) Barium (Ba) [26, 33]
(v) Manganese (Mn)	(v) Phosphorus (P)	(v) Cadmium (Cd) [26, 34, 35]
(vi) Molybdenum (Mo)	(vi) Potassium (K)	(vi) Lead (Pb) [26, 28, 36]
(vii) Nickel (Ni)	(vii) Selenium (Se)	(vii) Silver (Ag) [26, 37, 38]
(viii) Silicon (Si)	(viii) Sodium (Na)	(viii) Uranium (U) [26, 39, 40]
	(ix) Strontium (Sr)	
	(x) Sulphur (S)	
	(xi) Vanadium (Vn)	
	(xii) Zinc (Zn)	

**Table 2:** Characteristics of the participants.

	Number	Mean age (years)	Age range	Medication use (*n*)	Supplement use (*n*)
Gender					
Male	3	56.3	54–59	0	2
Female	3	36.6	30–45	3	0
Total	6	46.5	30–59	3	2

**Table 3:** Changes in element concentrations in distilled water after running the machine without feet.

Elements (ug/L)	Distilled water + salt (pre-FBS)	Distilled water + salt (post-FBS)	Mean difference	%change
Aluminum**^§^**	25.0	26.0	1.0	4.0
Antimony**^§^**	0.0	2.0	2.0	200.0
Arsenic**^§^**	0.0	6.0	6.0	600.0
Barium**^§^**	0.0	0.0	0.0	0.0
Boron**^†^**	0.0	1.0	1.0	100.0
Cadmium**^§^**	0.0	9.0	9.0	900.0
Calcium**^†^**	30.0	150.0	120.0	400.0
Chromium**^‡^**	4.0	23,634.0	23,630.0	590,750.0
Cobalt**^‡^**	0.0	320.0	320.0	320.0
Copper**^‡^**	40.0	280.0	240.0	600.0
Iron**^‡^**	31.0	116,421.0	116,390.0	375,451.6
Lead**^§^**	1.0	0.0	−1.0	−100.0
Lithium**^†^**	0.0	0.0	0.0	0.0
Magnesium	570.0	570.0	0.0	0.0
Manganese**^‡^**	0.0	1,566.0	1,566.0	1566.0
Molybdenum	50.0	3,155.0	3,105.0	6,210.0
Nickel**^‡^**	2.0	15,179.0	15,177.0	758,850.0
Phosphorus**^†^**	21.0	59.0	38.0	180.9
Potassium**^†^**	60.0	50.0	−10.0	−16.7
Selenium**^†^**	0.0	1.0	1.0	100.0
Silicon**^‡^**	20.0	1,170.0	1,150.0	5,750.0
Silver**^§^**	0.0	0.0	0.0	0.0
Sodium**^†^**	136,740.0	141,860.0	5,120.0	3.7
Strontium**^†^**	5.0	6.0	1.0	20.0
Sulfur	0.0	0.0	0.0	0.0
Uranium**^§^**	0.0	0.0	0.0	0.0
Vanadium ^†^	1.0	59.0	58.0	5,800.0
Zinc**^†^**	10.0	30.0	20.0	200.0

Total	**137,610.0**	**304,554.0**	**166,944.0**	**121.3**

Array component^‡^	**147.0**	**161,725.0**	**161,578.0**	**1,09,917.0**
Essential elements^†^	**137,437.0**	**142,786.0**	**5,349.0**	**3.9**
PTEs^§^	**26.0**	**43.0**	**17.0**	**65.4**

**^§^**PTEs: potentially toxic elements are defined to be aluminium, antimony, arsenic, barium, cadmium, lead, silver and uranium.

**^†^**Essential elements are defined to be boron, calcium, lithium, magnesium, phosphorus, potassium, selenium, sodium, strontium, sulphur, vanadium, and zinc.

**^‡^**Array component elements are to be chromium, cobalt, copper, iron, manganese, molybdenum, nickel, and silicon.

**Table 4:** Changes in element concentrations in tap water after running the machine without feet.

Elements (*μ*g/L)	Pre-FBS (*n* = 4)	Post-FBS (*n* = 6)	Post-FBS–Pre-FBS		*P *value
	Mean ± Std dev	Mean ± Std dev	Difference ± Std dev	%change	
Aluminum^§^	93.75 ± 11.35	105.00 ± 18.95	14.17 ± 12.22	15.1	0.257
Antimony^§^	**0.75 **± **0.50**	**1.83 **± **0.41**	**1.00 **± **0.63**	**133.3**	**0.038**
Arsenic^§^	**1.00 **± **0.00**	**5.50 **± **0.84**	**4.50 **± **0.84**	**450.0**	**0.010**
Barium^§^	20.00 ± 0.00	25.00 ± 5.48	5.00 ± 5.48	25.0	0.333
Boron^†^	35.00 ± 5.77	36.67 ± 5.16	3.33 ± 5.16	9.5	0.905
Cadmium^§^	**0.50 **± **1.00**	**6.50 **± **1.64**	**5.50 **± **2.43**	**1,100.0**	**0.010**
Calcium^†^	39,255.00 ± 1,354.51	39,843.33 ± 906.15	1,206.67 ± 893.37	3.1	0.609
Chromium^‡^	**3.50 **± **1.29**	**17,289.67 **± **4,240.36**	**17,286.67 **± **4,239.65**	**493,904.7**	**0.010**
Cobalt^‡^	**1.00 **± **0.00**	**249.17 **± **44.02**	**248.17 **± **44.02**	**24,816.6**	**0.010**
Copper^‡^	**465.00 **± **46.55**	**723.33 **± **93.31**	**253.33 **± **64.39**	**54.5**	**0.010**
Iron^‡^	**213.50 **± **183.72**	**88,689.17 **± **17,460.59**	**88,388.17 **± **17,532.11**	**41,399.6**	**0.010**
Lead^§^	2.75 ± 1.71	3.17 ± 1.17	0.33 ± 1.37	12.1	0.676
Lithium^†^	0.00 ± 0.00	1.17 ± 2.86	1.17 ± 2.86	0.0	0.800
Magnesium^†^	10,720.00 ± 437.34	11,025.00 ± 561.31	405.00 ± 427.73	3.7	0.476
Manganese^‡^	**5.25 **± **0.50**	**1,240.17 **± **212.04**	**1,235.00 **± **211.83**	**23,523.8**	**0.010**
Molybdenum^‡^	**47.50 **± **18.36**	**2,559.83 **± **440.07**	**2,505.17 **± **438.10**	**5,274.0**	**0.010**
Nickel^‡^	**3.25 **± **2.50**	**11,623.17 **± **2,076.03**	**11,621.00 **± **2,075.55**	**357,569.2**	**0.010**
Phosphorus^†^	16.75 ± 15.73	48.50 ± 28.03	37.33 ± 21.73	222.9	0.114
Potassium^†^	2,052.50 ± 235.28	2,146.67 ± 190.23	−11.67 ± 163.64	−0.6	0.610
Selenium^†^	0.75 ± 0.50	0.50 ± 0.55	0.00 ± 0.00	0.0	0.905
Silicon^‡^	**742.50 **± **120.93**	**1,805.00 **± **204.52**	**1,003.33 **± **170.37**	**135.1**	**0.010**
Silver^§^	0.00 ± 0.00	0.00 ± 0.00	0.00 ± 0.00	0.0	0.000
Sodium^†^	77,622.50 ± 41,701.87	101,911.67 ± 9,916.69	19,190.00 ± 38,454.88	24.7	0.114
Strontium^†^	200.50 ± 4.80	202.83 ± 7.88	3.17 ± 10.94	1.6	0.114
Sulfur^†^	6,245.00 ± 1,537.15	6,178.33 ± 1,234.35	−628.33 ± 1,253.99	−10.1	0.914
Uranium^§^	0.00 ± 0.00	0.00 ± 0.00	0.00 ± 0.00	0.0	0.000
Vanadium^†^	**1.00 **± **0.00**	**43.50 **± **11.71**	**42.50 **± **11.71**	**4,250.0**	**0.010**
Zinc^†^	22.50 ± 9.57	35.00 ± 17.61	16.67 ± 10.33	74.1	0.543

Total	**137,771.75 **± **43,704.77**	**285,799.67 **± **27,823.80**	**142,837.17 **± **36,748.30**	**103.7**	**0.010**

Array components^‡^	**1,481.50 **± **334.61**	**124,179.50 ** ± **24,547.41**	**122,540.83 **± **24,659.98**	**8,271.4**	**0.010**
Essential elements^†^	136,171.50 ± 43,506.28	161,473.17 ± 10,605.21	20,265.83 ± 39,878.15	14.9	0.171
PTEs^§^	118.75 ± 11.53	147.00 ± 25.42	30.50 ± 19.85	25.7	0.133

^§^PTEs: potentially toxic elements were defined to be aluminium, antimony, arsenic, barium, cadmium, lead, silver, and uranium.

^†^Essential elements were defined to be boron, calcium, lithium, magnesium, phosphorus, potassium, selenium, sodium, strontium, sulphur, vanadium, and zinc.

^‡^Array components were defined to be chromium, cobalt, copper, iron, manganese, molybdenum, nickel, and silicon.

Bold indicates a statistically significant difference, *P* < 0.05 (Mann-Whitney *U*-test).

**Table 5:** Changes in element concentrations in tap water after running the machine with participants feet.

Elements (*μ*g/L)	Pre-FBS (*n* = 6)	Post-FBS (*n* = 24)	Post-FBS FF–Pre-FBS		*P* value
	Mean ± Std dev	Mean ± Std dev	Difference ± Std dev	%change	
Aluminum^§^	110.80 **± **51.79	126.75 **± **44.05	5.00 **± **58.43	4.5	0.239
Antimony^§^	1.00 **± **1.41	1.71 **± **1.52	1.21 **± **1.50	120.8	0.056
Arsenic^§^	**1.00 ± 0.00**	**6.58 ± 1.02**	**5.58 ± 1.02**	**558.3**	**0.0001**
Barium^§^	**22.00 ± 4.47**	**26.04 ± 4.89**	**6.04 ± 4.89**	**27.5**	**0.034**
Boron^†^	40.00 **± **10.00	41.13 **± **6.31	4.04 **± **9.12	10.1	0.118
Cadmium^§^	**0.80 ± 1.10**	**8.54 ± 2.55**	**8.04 ± 2.80**	**1,005.2**	**< 0.0001**
Calcium^†^	39,316.00 **± **617.36	39,091.46 **± **1,198.06	−231.04 **± **1,354.99	−0.6	0.250
Chromium^‡^	**1.60 ± 2.07**	**23,548.21 ± 4,783.09**	**23,546.21 ± 4,782.52**	**1,471,638.0**	**< 0.0001**
Cobalt^‡^	**1.00 ± 0.00**	**332.05 ± 57.69**	**331.05 ± 57.69**	**33,105.0**	**< 0.0001**
Copper^‡^	**406.00 ± 190.34**	**836.71 ± 152.62**	**456.71 ± 318.01**	**112.5**	**< 0.0001**
Iron^‡^	**388.60 ± 178.33**	**114,629.83 ± 23,404.94**	**114,200.08 ± 23,447.22**	**29,387.6**	**< 0.0001**
Lead^§^	2.40 **± **1.14	2.50 **± **0.78	0.00 **± **0.78	0.0	0.607
Lithium^†^	1.40 **± **1.95	2.00 **± **2.30	0.25 **± **0.94	17.9	0.985
Magnesium^†^	10,647.60 **± **315.70	10,600.83 **± **398.56	−128.67 **± **509.11	−1.2	0.218
Manganese^‡^	**5.20 ± 0.45**	**1,643.83 ± 275.35**	**1,638.58 ± 275.26**	**31,511.2**	**< 0.0001**
Molybdenum^‡^	**48.00 ± 48.79**	**3,575.50 ± 569.09**	**3,515.50 ± 567.16**	**7,324.0**	**< 0.0001**
Nickel^‡^	**2.00 ± 2.12**	**15,946.42 ± 2,770.42**	**15,943.92 ± 2,769.51**	**797,195.8**	**< 0.0001**
Phosphorus^†^	**26.60 ± 9.81**	**70.29 ± 26.79**	**44.29 ± 26.82**	**166.5**	**0.004**
Potassium^†^	**2,118.00 ± 132.36**	**2,586.71 ± 171.82**	**489.21 ± 162.18**	**23.1**	**< 0.0001**
Selenium^†^	**0.22 ± 0.44**	**1.04 ± 0.69**	**0.77 ± 0.64**	**348.5**	**0.032**
Silicon^‡^	**634.00 ± 217.55**	**1,933.38 ± 250.56**	**1,338.38 ± 238.36**	**211.1**	**< 0.0001**
Silver^§^	0.00	0.00	0.00	0.0	0.000
Sodium^†^	**96,458.00 ± 8,298.75**	**95,941.42 ± 8,473.96**	**−1,958.58 ± 6,064.23**	**−2.0**	**< 0.0001**
Strontium^†^	204.80 **± **14.24	190.83 **± **12.16	−11.67 **± **9.95	−5.7	0.070
Sulfur^†^	9,858.00 **± **2,707.58	8,685.71 **± **1,471.65	−496.79 **± **2,448.86	−5.0	0.512
Uranium^§^	0.20 **± **0.45	0.29 **± **0.46	0.04 **± **0.36	20.8	0.851
Vanadium^†^	**0.60 ± 0.55**	**59.96 ± 12.70**	**59.04 ± 12.66**	**9,840.3**	**< 0.0001**
Zinc^†^	**18.00 ± 8.37**	**34.13 ± 8.78**	**16.63 ± 7.65**	**92.4**	**0.001**

Total	**160,313.82 ± 7,685.23**	**319,923.84 ± 37,781.00**	**158,783.82 ± 34,556.21**	**99.0**	**< 0.0001**

Array components^‡^	**1,486.40 ± 7,702.91**	**160,512.55 ± 31,630.27**	**160,970.43 ± 31,896.70**	**10,829.5**	**< 0.0001**
Essential elements^†^	158,689.22 **± **368.55	159,238.88 **± **9,413.96	−2,212.53 **± **8,230.33	−1.4	0.8013
PTEs^§^	**138.20 ± 49.37**	**172.42 ± 41.38**	**25.92 ± 58.46**	**18.8**	**0.0423**

^§^PTEs: potentially toxic elements were defined to be aluminium, antimony, arsenic, barium, cadmium, lead, silver, and uranium.

^†^Essential elements were defined to be boron, calcium, lithium, magnesium, phosphorus, potassium, selenium, sodium, strontium, sulphur, vanadium, and zinc.

^‡^Array components were defined to be chromium, cobalt, copper, iron, manganese, molybdenum, nickel, and silicon.

Bold indicates a statistically significant difference, *P* < 0.05 (Mann-Whitney *U* test).

**Table 6:** Summary of differences between element concentrations after footbath runs with feet and without feet.

Elements (*μ*g/L)	Post-FBS–Pre-FBS no feet	Post-FBS–Pre-FBS with feet	*P *value
	Mean ± Std dev	Mean ± Std dev	
Aluminum^§^	14.17 **± **12.22	5.00 **± **58.43	0.487
Antimony^§^	1.00 **± **0.63	1.21 **± **1.50	0.8859
Arsenic^§^	**4.50 ± 0.84**	**5.58 ± 1.02**	**0.016**
Barium^§^	5.00 **± **5.48	6.04 **± **4.89	0.911
Boron^†^	3.33 **± **5.16	4.04 **± **9.12	0.814
Cadmium^§^	5.50 **± **2.43	8.04 **± **2.80	0.064
Calcium^†^	**1,206.67 ± 893.37**	**−231.04 ± 1,354.99**	**0.02**
Chromium^‡^	**17,286.67 ± 4,239.65**	**23,546.21 ± 4,782.52**	**0.003**
Cobalt^‡^	**248.17 ± 44.02**	**331.05 ± 57.69**	**0.001**
Copper^‡^	253.33 **± **64.39	456.71 **± **318.01	0.162
Iron^‡^	**88,388.17 ± 17,532.11**	**114,200.08 ± 23,447.22**	**0.008**
Lead^§^	0.33 **± **1.37	0.00 **± **0.78	0.909
Lithium^†^	1.17 **± **2.86	0.25 **± **0.94	0.994
Magnesium^†^	**405.00 ± 427.73**	**−128.67 ± 509.11**	**0.024**
Manganese^‡^	**1,235.00 ± 211.83**	**1,638.58 ± 275.26**	**0.001**
Molybdenum^‡^	**2,505.17 ± 438.10**	**3,515.50 ± 567.16**	**0.001**
Nickel^‡^	**11,621.00 ± 2,075.55**	**15,943.92 ± 2,769.51**	**0.001**
Phosphorus^†^	37.33 **± **21.73	44.29 **± **26.82	0.502
Potassium^†^	**−11.67 ± 163.64**	**489.21 ± 162.18**	**< 0.0001**
Selenium^†^	**0.00 ± 0.00**	**0.77 ± 0.64**	**0.010**
Silicon^‡^	**1,003.33 ± 170.37**	**1,338.38 ± 238.36**	**0.003**
Silver^§^	0.00	0.00	
Sodium^†^	**19,190.00 ± 38,454.88**	**−1,958.58 ± 6,064.23**	**0.016**
Strontium^†^	**3.17 ± 10.94**	**−11.67 ± 9.95**	**0.009**
Sulfur^†^	−628.33 ± 1,253.99	−496.79 ± 2,448.86	0.490
Uranium^§^	0.00 **± **0.00	0.04 **± **0.36	0.731
Vanadium^†^	**42.50 ± 11.71**	**59.04 ± 12.66**	**0.005**
Zinc^†^	16.67 **± **10.33	16.63 **± **7.65	0.956

Total	142,837.17 **± **36,748.30	158,783.82 **± **34,556.21	0.2962

Array components^‡^	**122,540.83 ± 24,659.98**	**160,970.43 ± 31,896.70**	**0.0051**
Essential elements^†^	20,265.83 **± **39,878.15	−2,212.53 **± **8,230.33	0.1011
PTEs^§^	30.50 **± **19.85	25.92 **± **58.46	0.8697

^§^PTEs: potentially toxic elements were defined to be aluminium, antimony, arsenic, barium, cadmium, lead, silver, and uranium.

^†^Essential elements were defined to be boron, calcium, lithium, magnesium, phosphorus, potassium, selenium, sodium, strontium, sulphur, vanadium, and zinc.

^‡^Array components were defined to be chromium, cobalt, copper, iron, manganese, molybdenum, nickel, and silicon.

Bold indicates a statistically significant difference, *P* < 0.05 (Mann-Whitney *U-*test).

**Table 7:** Composition of grade 316 stainless steel.

Element	Percentage composition
Chromium	16–18%
Nickel	10–14%
Molybdenum	2-3%
Manganese	2%
Silicon	1%
Carbon	0.08%
Phosphorus	0.045%
Sulfur	0.03%

[47]
